# Biotechnology-assisted cancer therapy using metal sulfides based on their optical and thermophysical properties

**DOI:** 10.1039/d4na00929k

**Published:** 2025-03-21

**Authors:** Fei Luo, Shaohua Song, Gang Zhou, Youfu Wang, Zhiren Fu, Hao Liu

**Affiliations:** a Department of General Surgery, Ruijin Hospital, Shanghai Jiao Tong University, School of Medicine Shanghai 200025 P. R. China haoliu6@126.com; b College of Materials, Shanghai Dianji University Shanghai 201306 P. R. China; c Key Laboratory of Integrated Regulation and Resources Development on Shallow Lakes, Ministry of Education, College of Environment, Hohai University Nanjing 210098 P. R. China; d School of Chemistry and Chemical Engineering, Frontiers Science Center for Transformative Molecules, Shanghai Jiao Tong University Shanghai 200240 P. R. China

## Abstract

Two-dimensional transition metal sulfides (2D-TMSs) have received considerable attention in recent years owing to their exceptional features and diverse applications. Two-dimensional nanostructures of transition metal sulfides exhibit highly anisotropic properties, excellent mechanical strength, biocompatibility, a large surface area, and the ability to enhance functionality through surface modification methods. These features make them an ideal and attractive material for developing multifunctional platforms. In this review, we provide a comprehensive introduction to various configurations of nanostructures based on 2D-TMSs, including their modified structures such as vacancies and nanoflowers, as well as their composites, which encompass doped structures, alloyed structures, particles/dots on sheets, 2D-TMS-based heterojunctions, and core–shell nanostructures. This chemistry and configuration of 2D-TMSs have captured the attention of many researchers, driving them to delve into the diverse applications of these materials in the biomedical field, especially in drug delivery, photothermal therapy, sonodynamic therapy, and ferroptosis. Finally, the review summarizes the opportunities, challenges, and prospects of 2D-TMSs, emphasizing their crucial role in shaping the future of technology, medicine, and cancer therapy. The distinctive properties of 2D-TMSs make them promising contenders for various applications, and their continued exploration holds tremendous potential for scientific and technological progress.

## Abstract 2

This review provides a comprehensive introduction to 2D-TMSs, including their nanostructures, performance, and applications in drug delivery, photothermal therapy, sonodynamic therapy, and ferroptosis.

## Graphical abstract

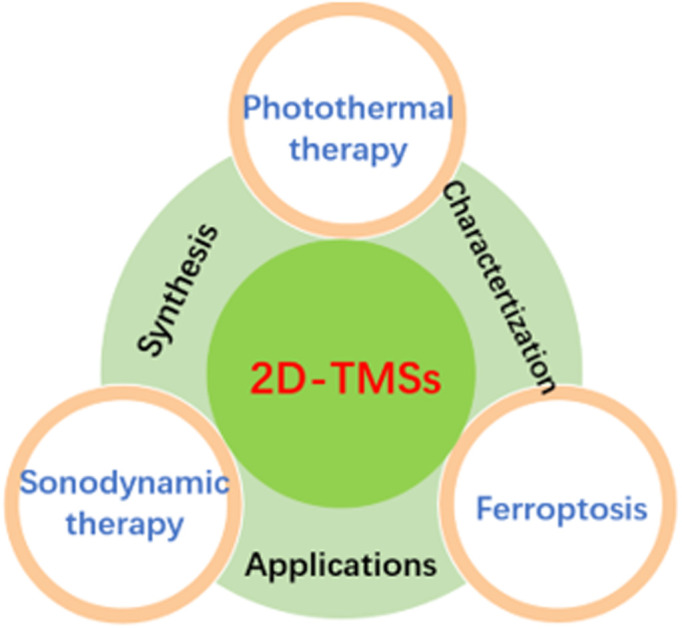

## 1. Introduction

**Fig. 1:**
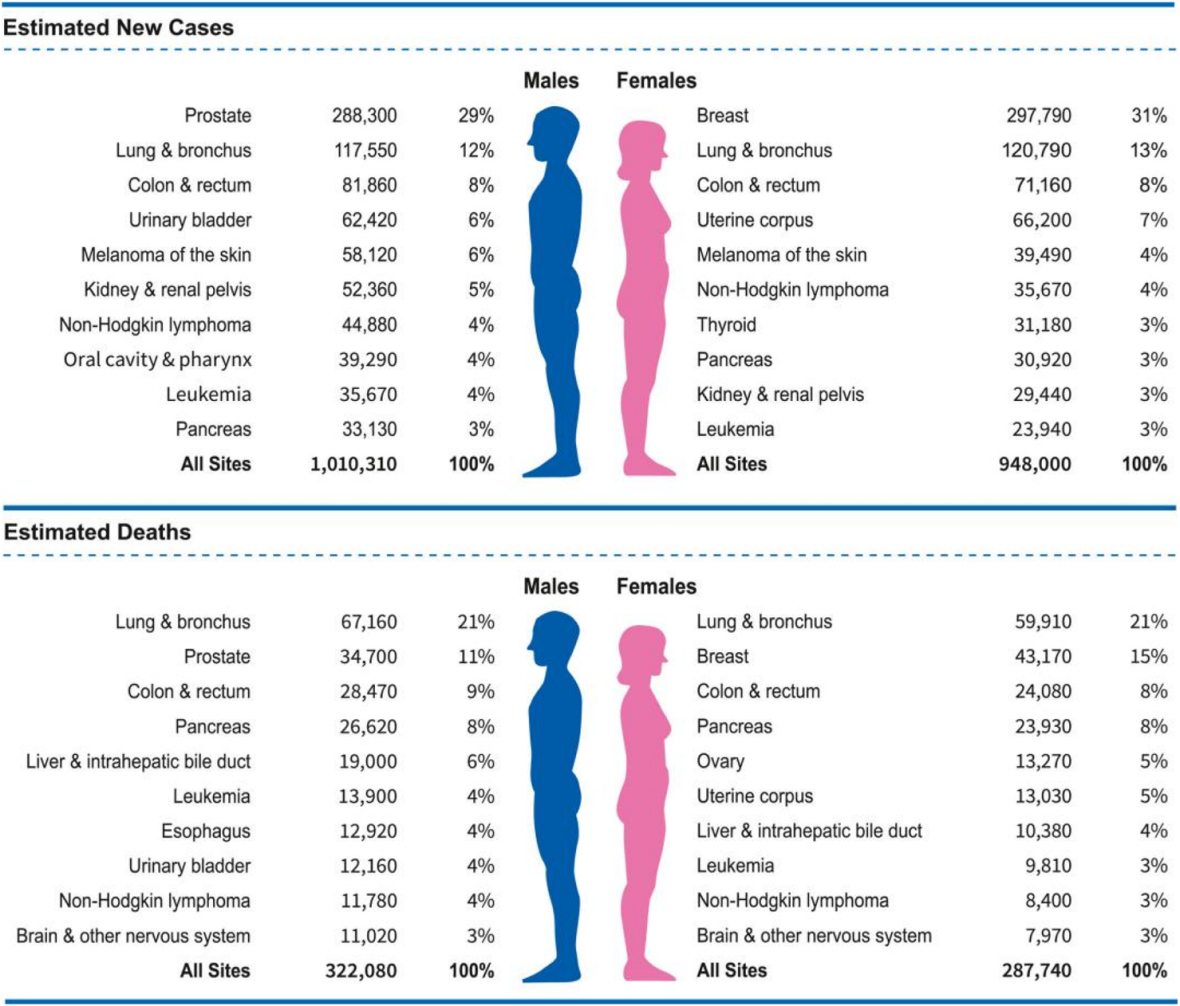
Statistics on cancer deaths in 2020 and predicted new cancer cases in 2023.^6^

Cancer is a major health concern, affecting approximately 180 out of every 100 000 individuals.^1^ It is now considered a prevalent and increasingly common disease, with a rising incidence rate.^2^ Efforts to develop new cancer treatments are being made worldwide; however, the currently available methods are limited, and a definitive cure remains elusive, resulting in a high mortality rate.^2,3^ Treating cancer requires advanced medical technology and substantial financial and temporal investment, as well as entails significant side effects.^4,5^ Consequently, there is an urgent need to develop novel cancer treatment strategies. Recently, the latest Cancer Statistics for 2023 have been published, detailing cancer mortality data from 2020 and estimating the incidence of new cancer cases in 2023 (Fig. 1).^6^

In recent years, significant advancements have been made in the development of various anticancer drugs and technologies. While chemotherapy drugs remain the primary choice for clinical cancer treatment, their non-specific distribution in the body often leads to severe toxic effects.^7^ The constant changes and spread of tumor cells pose challenges in observing and diagnosing cancer lesions, limiting the effectiveness of drug chemotherapy. Traditional nanomaterial delivery systems based on non-covalent physical packaging suffer from issues such as low drug loading and drug leakage due to the poor affinity between the drug and the carrier.^7,8^ Therefore, there is a pressing need to develop new nanomaterials to enhance the effectiveness of cancer treatment and mitigate adverse effects.^9^

Two-dimensional transition metal sulfides (2D-TMSs) have garnered substantial research interest due to their high specific surface area, tunable interlayer spacing, surface functional chemical properties, and intrinsic optical characteristics.^10–12^ These metal sulfides have been extensively studied for applications in photodynamic therapy, photothermal therapy, sonodynamic therapy, and ferroptosis.^13^ Notably, various 2D-TMSs, for example, MoS_2_, Ag_2_S, WS_2_, and VS_2_ have been researched for anti-cancer applications due to their structural and optical characteristics.^10,14,15^ Regarding optical properties, 2D-TMSs provide exceptional optical stability and remarkable efficiency in converting light into heat energy. Additionally, they exhibit exceptional efficiency in absorbing near-infrared (NIR) radiation in the wavelength range spanning from 700 to 1100 nanometers, which is an essential requirement for *in vivo* applications such as photodynamic therapy and photothermal therapy.

Photodynamic therapy (PDT) is a safe and non-invasive treatment that utilizes photosensitizers and light activation.^16^ When tumor sites are irradiated with specific wavelengths of light, photosensitive drugs selectively retained within the tumor tissue are activated, leading to photochemical reactions in an aerobic environment and resulting in necrosis of the tumor tissue.^17^ PDT offers specific advantages, including reducing cumulative tumor toxicity, avoiding multidrug resistance, promoting the immune response of the tumor, and enabling long-term tumor ablation.^18–20^

Photothermal therapy (PTT) has gained significant attention in the medical field as a new treatment technology.^21,22^ PTT is a local thermal ablation method that utilizes photothermal agents, for instance, compounds such as ICG, porphyrins, and gold nanostructures are employed to induce protein denaturation and cell membrane rupture in target cells or tissues, primarily tumors, under light irradiation.^23,24^ This minimally invasive cancer therapy provides enhanced selectivity and minimal side effects, improving tumor treatment.^25,26^ When exposed to near-infrared laser radiation, photothermal materials introduced into cancer cells or tumor tissues produce a significant quantity of oxygen-free radicals. This results in localized heating of the cancer cells and tumor tissues, enabling precise ablation and elimination of tumor cells.^27,28^ Tumor tissues are more susceptible to heat damage due to differences in vasculature between tumor blood vessels and normal tissue blood vessels.^21^ Protein denaturation and activation and inactivation of downstream pathways contribute to the decrease in tumor cell viability after hyperthermia.^29,30^ The use of 2D-TMSs in combination with PTT has been extensively studied.^31–33^ Moreover, the precise targeting of treatment is crucial to minimize damage to surrounding healthy tissues.^34,35^ 2D-TMSs, through their imaging capabilities such as photoacoustic imaging (PAI), computed tomography imaging (CT), and nuclear magnetic resonance imaging (MR), can accurately determine the location of cancer and improve the therapeutic effect of PPT.^36,37^

Sonodynamic therapy (SDT) has emerged as a groundbreaking and highly promising noninvasive methodology, drawing inspiration from PDT. The differentiation between SDT and PDT is based on the energy source utilized to initiate the activation of the sensitizers, with SDT utilizing ultrasound and PDT using light. The limited light penetration depth poses a challenge for PDT in treating deep-seated tumors. However, SDT offers a significant advantage as ultrasound can be precisely concentrated, penetrating soft tissue to depth of several tens of centimeters.^38^ Due to the high penetration of ultrasound, SDT is superior to photodynamic therapy for treating deep-seated tumors. Addressing the main limitation of PDT, this characteristic of SDT relies on the simultaneous combination of low-intensity ultrasound, molecular oxygen, and a sonosensitizer to generate reactive oxygen species (ROS) for its efficacy.^39^ SDT, as an innovative treatment modality, has shown promising outcomes with significant anticancer effects observed in both *in vitro* and *in vivo* studies.^40^

Ferroptosis is an iron-dependent, non-apoptotic mechanism of cell death that relies on ROS generated through the Fenton reaction to trigger phospholipid peroxidation in plasma membranes.^41^ This type of cellular death is triggered by the dysregulation of lipid peroxidation (LPO). Key regulators such as glutathione peroxidase 4 (GPX4) and the antioxidant glutathione (GSH) play crucial roles in safeguarding cells against lipid peroxidation and thwarting ferroptosis.^42^ Either the depletion of glutathione (GSH) or the down-regulation of GPX4 can lead to increased LPO. Cancer cells, in comparison to normal cells, display an altered intracellular redox state characterized by elevated levels of antioxidants, including GSH. Intracellular ferric iron can decrease GSH levels, whereas ferrous ions engage in the Fenton reaction with hydrogen peroxide (H_2_O_2_), leading to increased hydroxyl radical levels to advance ferroptosis.^43^ As a result, strategies to increase the iron reservoir and exhaust GSH have emerged as a promising approach to trigger ferroptotic cell death. Moreover, ROS produced due to iron metabolism play a vital role in accelerating the accumulation of LPO during ferroptosis. This process is significant because ferroptosis offers an effective alternative to traditional apoptosis,^44^ which may be resistant to frequent treatments. Therefore, the regulation of essential factors, including GPX4, GSH and iron metabolism, is pivotal for promoting and accelerating the ferroptosis cell death pathway. Ferroptosis in cancer cells revolves around three cellular pathways: (i) iron metabolism, leading to accumulated iron; (ii) diminished antioxidant defense through the GPX4/GSH pathway; and (iii) metabolism of amino acids. Additionally, lipid peroxidation pathways, mediated by mitochondrial voltage-dependent anion channels (VDACs) and the p53 gene, play a role in influencing ferroptosis. The prolonged initiation of lipid peroxidation results from accumulated iron. When antioxidants, particularly those involved in the GPX4/GSH pathway, are depleted, the structural integrity of the cell membrane collapses, ultimately leading to cell demise.^45^ Therefore, a more comprehensive understanding of ferroptosis can aid researchers in developing novel cancer treatments and medications.

This review extensively examines the preparation of various 2D-TMS composite materials, with a particular focus on their structural modifications and surface properties for cancer treatment. It also discusses the applicability of these nanocomposite materials across multiple domains of cancer therapy, including PDT, PTT, SDT, and ferroptosis, highlighting significant advancements and recent breakthroughs in this field. Initially, this review emphasizes the importance of the configuration and properties of 2D-TMSs, detailing their composition and characteristics. Subsequently, it classifies and presents interesting nanostructures based on 2D-TMSs, outlining their synthesis methods and the key reasons for their development. Furthermore, the review thoroughly explores the potential of these nanocomposite materials in advancing drug delivery and tumor treatment. Finally, it concludes with an insightful outlook that summarizes the key findings.

## 2. Energy band theory and brief principles of ROS production

According to energy band theory, the band potential assumes a pivotal role in regulating the band structure and governing the migration of internal carriers to the catalyst surface. The primary determinants of the activity of catalysts in catalyzing specific chemical reactions are the energy band alignments, specifically the valence and conduction band positions. The potential can be changed by the formed heterojunction, playing a pivotal role in determining the energy states within the valence band (VB) and conduction band (CB). To elaborate, when a 2D-TMS is combined with another compound, the band bends and rearranges. This phenomenon facilitates charge exchange at the interface, enabling the efficient catalysis of redox reactions. Thus, photogenerated carriers travel along specific channels and carry out redox reactions.

ROS-based therapy mainly includes PDT and SDT. PDT utilizes photosensitizers to generate toxic singlet oxygen under light excitation. Photosensitizers accumulate in tumor tissues, and the subsequent light excitation process promotes ROS generation, leading to the death of tumor cells.^46^ In principle, the photosensitized (excited) photosensitizer can directly react with suitable substrates (unsaturated fats, proteins, or nucleic acids), generating unstable free radicals through the transfer of either protons or electrons, known as a type I reaction. In the presence of oxygen, this results in the formation of oxygen-containing products, for example, superoxide anion radicals (˙O_2_^−^), hydroxyl radicals (˙OH), or hydrogen peroxide (H_2_O_2_). Conversely, the photosensitizer, in its excited state, has the capability to undergo reactions with molecular oxygen, generating singlet oxygen (^1^O_2_) *via* energy transfer, known as a type II reaction. Under high oxygen content conditions, ^1^O_2_ is the main cytotoxin in PDT.^47,48^ While the balance between type I and type II reactions is contingent upon the type and concentration of the photosensitizer, oxygen levels, and the extent of irradiation, the detailed mechanisms underlying the generation of reactive ROS and tumor ablation in PDT are not completely understood. In contrast, for deeper tumors, SDT utilizes sonosensitizers to convert oxygen into ROS under ultrasound stimulation. Thanks to the high penetration ability of ultrasound, SDT is superior to PDT in treating deep tumors.^49^ A substantial volume of research is currently dedicated to exploring the relationship between ROS and SDT, resulting in a wealth of significant findings within this domain. Researchers have systematically developed and designed inorganic nano-agents for ultrasound sensitization based on the principles of SDT. Upon absorbing energy, sonosensitizers based on conductive materials generate electron–hole pairs, initiating a sequence of reactions that result in the production of ROS, thereby achieving the SDT effect.^50^ TMS-based nano-sensitizers, due to their suitable bandgap and susceptibility to light or ultrasound excitation, have progressively shifted from photocatalysis to biomedical contexts. Through the transfer of ultrasound energy to these sonosensitizers, electron–hole pairs are generated. Subsequently, unbound electrons engage in reactions with oxygen and various other molecules, leading to ROS generation and subsequent cell demise. Nevertheless, maintaining the CB and VB within optimal ranges is imperative to ensure effective ROS generation under ultrasound.^51^ Furthermore, the sonosensitizers must possess an appropriate bandgap width and facilitate ROS production *via* a series of redox reactions initiated by ultrasound stimulation. Unlike PDT and SDT, CDT relies on *in vivo* Fenton or Fenton-like reactions, where H_2_O_2_ in the tumor microenvironment reacts with an external catalytic agent to generate hydroxyl radicals (˙OH).^52^ The commonality among these ROS-based therapies is the production of a substantial quantity of ROS in tumor tissues, triggering oxidative stress and inducing cell death.

**Fig. 2:**
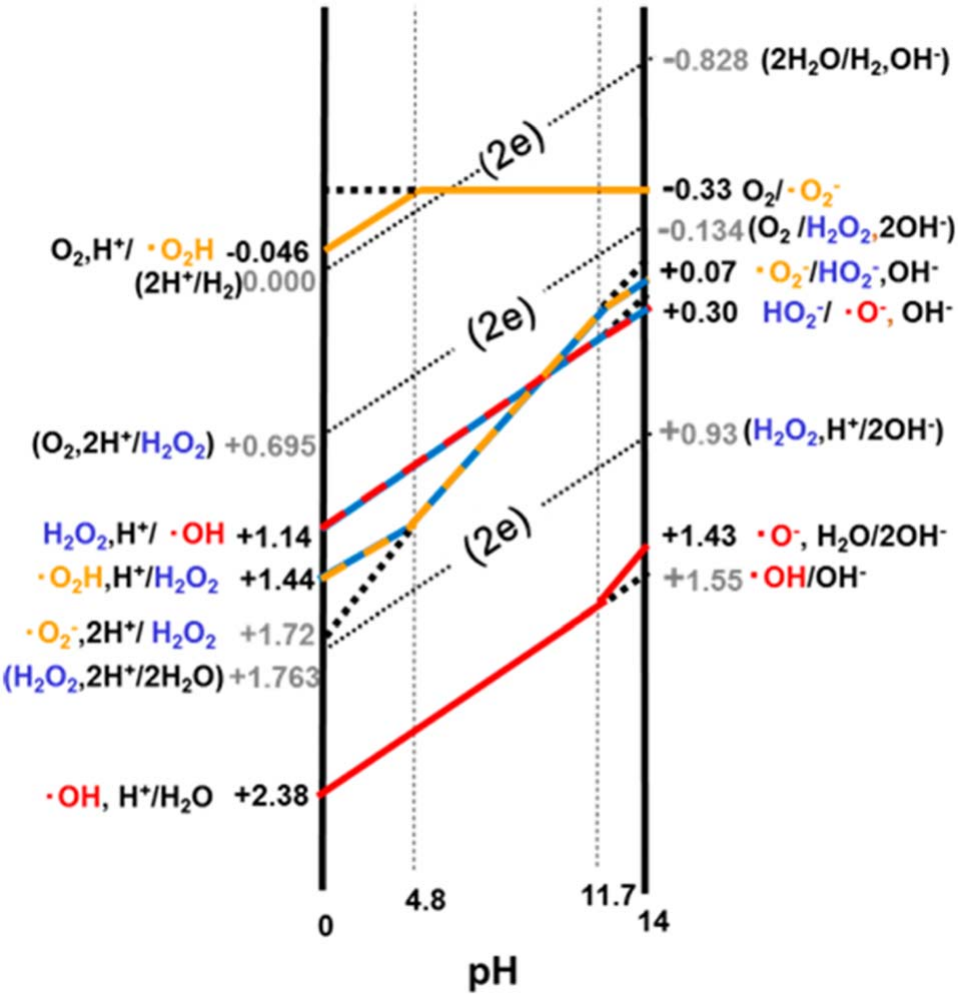
The pH dependence of one-electron redox reactions involving H_2_O (water), H_2_O_2_ (hydrogen peroxide), and O_2_ (oxygen). The dotted line in the graph indicates a two-electron (2e) process.^54^

ROS (˙O_2_^−^, H_2_O_2_, ˙OH, and ^1^O_2_) can be generated sequentially from both molecular oxygen (O_2_) and water (H_2_O);^53^ the relative pH dependence of redox reactions involving H_2_O, H_2_O_2_, and O_2_ is shown in Fig. 2.^54^ They serve as signaling molecules within cells but are also considered as inevitable toxic byproducts of aerobic metabolism.^55^ ROS play a crucial role in maintaining the balance of oxidation and reduction within tumor tissues, rendering them a crucial factor in tumor therapy. Typically, oxidative stress disrupts the equilibrium within tumors due to the excessive accumulation of ROS, ultimately inducing programmed cell death and necrosis in cancer cells.^56^ As nanomedicine advances within the realm of cancer treatment, recent innovations in cancer treatments based on ROS have been propelled by a variety of nanomaterials and nanotechnologies.^57,58^ Research has shown that elevated ROS levels are present in all cancer cells and are closely linked to the development of neoplasms.^59,60^ However, this occurrence creates a biological divergence between the role of ROS in carcinogenesis and our conventional understanding that elevated levels of ROS in tumor sites lead to oxidative damage in cancer cells. Therefore, investigating the role of ROS in tumorigenesis and aggressiveness is important for advancing therapeutic strategies.^57^ Enhanced antioxidative defense mechanisms are observed in cancer cells with accumulated overexpressed ROS, altering the redox homeostasis from its normal state to a new equilibrium characterized by increased rates of ROS generation and scavenging. This adaptation ultimately ensures that ROS levels in cancer cells remain below the toxic threshold.^61^ The activation of particular redox-sensitive transcription factors such as Nf-κB and Nrf2, along with the overexpression of specific redox substances like GSH and superoxide dismutase, constitutes the endogenous antioxidative defense mechanisms. These substances work together to mitigate the cytotoxic effects of heightened ROS in tumor sites, aiding cancer cells in evading oxidative stress damage.^62^ Consequently, cancer cells rely significantly on their inherent antioxidative defense systems and exhibit heightened susceptibility to externally induced ROS generation, for example, dietary components, radiation, pharmaceuticals, and more.^63^

## 3. Photodynamic therapy of transition metal sulfides

In recent years, 2D-TMSs have garnered significant attention due to their exceptional physical, chemical, and morphological properties. When 2D-TMSs are employed in cancer therapy, they need to possess favorable characteristics such as good compatibility, excellent dispersion, prevention of aggregation, consistent physiological robustness and diminutive particle dimensions. Liquid-mediated and solvent-thermal/hydrothermal methods have become favored options for synthesizing 2D-TMSs among existing techniques, as they partially fulfill these criteria. Nevertheless, variations in layer thickness, dispersion and morphology may be observed among products designed using different synthetic methods.

Although hydrothermal/solvothermal methods are simple and environmentally friendly for synthesizing 2D-TMSs, challenges still remain in controlling their morphology. Additionally, it is often difficult to remove surface-deposited functional molecules, including substances like oleylamine, oleic acid, and other coating agents, and there is a potential for nanosheet aggregation in certain instances. Since these molecules are hydrophobic, further modifications are essential to overcome these limitations and enhance the applicability of these structures in biomedicine. It is worth noting that, compared to exfoliation methods, hydrothermal/solvothermal methods have more advantages in the synthesis of unique or composite nanostructures. Hydrothermal/solvothermal process-based methods often enable one-step synthesis, streamlining operational procedures significantly. This suggests substantial potential for developing multifunctional platforms for cancer diagnosis and treatment.

Therefore, despite some challenges, hydrothermal/solvothermal methods remain an effective approach for preparing 2D-TMSs, especially when the synthesis of special or composite structures is required. Through optimizing synthesis conditions and subsequent modifications, some challenges can be overcome to ensure that the obtained products have the desired morphology, dispersion, and biocompatibility. The potential applications of these methods in constructing cancer treatment and diagnosis platforms are highly anticipated.

### 3.1 PDT and PTT of MoS_2_

**Fig. 3:**
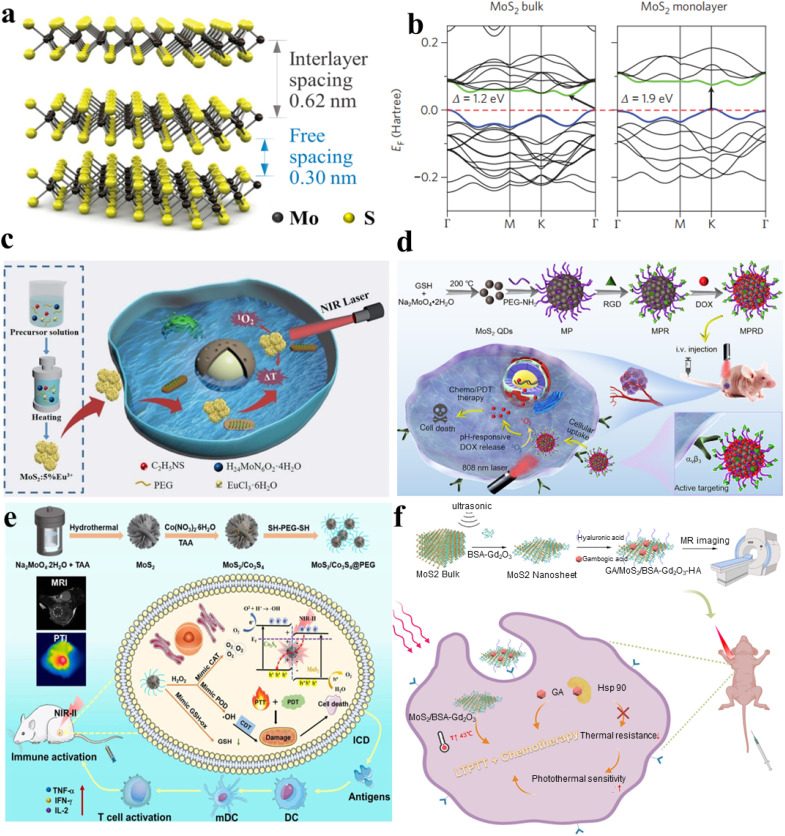
(a) A 3D schematic depiction of a standard MX_2_ structure, where chalcogen atoms (X) are depicted in yellow, and the metal atoms (M) are illustrated in grey.^64^ (b) Electronic traits and transport behaviors in transition metal dichalcogenides (TMDCs), featuring band structures obtained through first-principles density functional theory calculations for both bulk and monolayer MoS_2_ and WS_2_.^66^ (c) The synthetic pathway of MoS_2_:5% Eu^3+^ and its utilization in the integrated treatment of PDT and PTT.^72^ (d) Schematic depiction of the manufacturing process for MPRD and its application in combined chemo/PDT guided by fluorescence imaging.^73^ (e) Schematic representation of MoS_2_/Co_3_S_4_@PEG + NIR-II preparation and its anticancer process.^74^ (f) Diagram outlining the exfoliation and preparation procedures for GA/MoS_2_/BSA-Gd_2_O_3_-HA in the context of MRI-guided combined LTPTT and chemotherapy.^75^

2D-TMSs, such as MoS_2_ (molybdenum disulfide), have garnered more attention for cancer treatment compared to one-dimensional materials due to their ability to form complex structures and their wider range of applications. MoS_2_ is a member of the layered transition-metal dichalcogenide family, and its crystals consist of vertically stacked layers that are weakly interacting and bound through van der Waals forces (Fig. 3a).^64^ The materials exhibit layered structures with the X–M–X arrangement, where chalcogen atoms are positioned in two hexagonal planes, separated by a plane containing metal atoms.^65^ The electronic properties and transport of these materials, including MoS_2_, have been thoroughly investigated using first-principles density functional theory (DFT) calculations to analyze the band structures in both bulk and monolayer states. The first-principles calculations reveal the band structures of both bulk and monolayer MoS_2_, as shown in Fig. 3b.^66^ Its small bandgaps enable NIR absorption. The NIR absorption capacity and exceptional surface area of MoS_2_ in its two-dimensional form (2D-MoS_2_) and its nanocomposites have spurred extensive investigation in the field of cancer treatment. PTT uses the thermal energy generated from NIR light (700–1400 nm) absorbed by two-dimensional materials to induce hyperthermia or thermal ablation, effectively eradicating tumor cells.^67^ Consequently, PTT offers unparalleled advantages in cancer treatment, including lower costs, enhanced targeting selectivity, high anti-cancer efficacy,^68^ and minimal side effects.^69^ Additionally, by combining PTT with PDT, photosensitizers generate ROS and activate tumor cells using specific wavelengths of light to eliminate them.^70^ The combination of PTT and PDT has the potential to enhance therapeutic effects and improve photothermal conversion efficiency (PCE).^71^

For example, Zhou *et al.* synthesized MoS_2_:5% Eu^3+^ nanoflowers through employing a bottom–up hydrothermal technique, where Eu^3+^ ions were seamlessly integrated into MoS_2_ nanosheets. The resulting MoS_2_:5% Eu^3+^ nanocomposite displayed proficient utilization of NIR light, accompanied by a remarkable photothermal conversion efficiency (PCE), as evident from the spectra of UV-visible-near infrared (UV-vis-NIR) absorption. The integration of Eu^3+^ ions resulted in a notable improvement, with MoS_2_:5% Eu^3+^ achieving a PCE of approximately 49.05%, surpassing pristine MoS_2_ by 1.75 times. They proposed a synergistic approach involving PDT and PTT utilizing the MoS_2_:5% Eu^3+^ nanocomposite (Fig. 3c).^72^ The MoS_2_ doped with 5% Eu^3+^ serves as an efficient photosensitizer in cancer therapy. Under 808 nm laser irradiation, it acts as an effective light absorber for PTT and induce the generation of cytotoxic ROS, facilitating PDT for cancer treatment. The efficacy of *in vitro* breast cancer treatment is convincingly demonstrated through the synergistic application of combined PTT and PDT. Capitalizing on this unique nanostructure, the MoS_2_:5% Eu^3+^ nanomaterials demonstrated enhanced NIR absorption, higher ROS generation and improved biocompatibility, establishing them as ideal photothermal agents for the synergistic combination of PDT and PTT.

With the advent of nanotechnology, a diverse array of quantum dots (QDs), spanning types like graphene QDs and graphitic carbon nitride QDs, have been harnessed as versatile nanoplatforms. These nanomaterials serve for concurrent fluorescence imaging and effective cancer therapy; these QDs, characterized by their vivid photoluminescence, favorable biocompatibility, and swift cellular uptake, are employed. Li *et al.* successfully engineered a multifunctional therapeutic diagnostic nanoplatform named “MPRD” using MoS_2_ quantum dots (Fig. 3d).^73^ The PEGylated MoS_2_ quantum dots (MP) underwent covalent attachment of the targeting moiety RGD peptide, yielding the RGD-conjugated MP (MPR). Subsequently, doxorubicin (DOX), an antitumor drug, was loaded onto MPR to form MPRD. The MoS_2_ quantum dots in the MPRD possess inherent ROS generation ability, demonstrating exceptional performance in PDT when exposed to 808 nm NIR laser irradiation *in vivo*. Critically, when the MPRD is functionalized with the RGD peptide, it gains the ability to specifically target and penetrate tumor cells expressing αvβ3 integrin, which is facilitated through receptor-mediated endocytosis involving the αvβ3 receptor. This specificity allows for the regulated release of the chemotherapeutic payload, DOX, triggered by the intracellular acidic pH of tumor cells. The real-time tracking of DOX release is accomplished by monitoring the heightened dual-channel fluorescence signals emitted individually by DOX and MoS_2_ QDs. By harnessing the capabilities of fluorescence imaging, a collaborative approach is employed for tumor-targeted chemotherapy and PDT, proving effective in restricting tumor growth in mice bearing tumors. The integration of PDT and targeted chemotherapy in MPRD, guided by fluorescence imaging, demonstrates a promising strategy for achieving enhanced therapeutic outcomes in cancer treatment. The research introduces MoS_2_ QDs as a highly targeted theragnostic nanoplatform, showcasing their potential in guiding combinational chemo/PDT strategies through fluorescence imaging.

Efficient strategies for ensuring an adequate supply of oxygen are crucial for cancer therapy targeting hypoxia. Considering the abundance of aqueous environments within living organisms, one promising approach is photocatalytic oxygen generation *via* water-splitting is viewed as an effective strategy for replenishing oxygen. Kang *et al.* utilized MoS_2_/Co_3_S_4_@PEG nanoflowers, denoted as MSCs@PEG, to reveal near-infrared II (NIR-II) induced oxygen generation for targeted tumor therapy in hypoxic environments (Fig. 3e). Initially, MoS_2_ nanoflowers are fabricated using a hydrothermal synthesis approach, after which Co_3_S_4_ nanodots are deposited onto their surface to form a heterostructure. MSCs@PEG demonstrate outstanding absorption of NIR-II light and remarkable photothermal conversion efficiency (39.8%, 1064 nm). Furthermore, hyperpyrexia is pivotal in providing additional energy for the simultaneous excitation of MoS_2_ (1.14 eV) and Co_3_S_4_ (1.40 eV) under NIR-II illumination (1064 nm). Examination of the energy band structures reveals the Z-scheme mechanism inherent in the nanomaterial of MSCs, showcasing its resilient redox capabilities for water oxidation, leading to the concurrent generation of ROS and oxygen. Moreover, MSCs@PEG also exhibit peroxidase (POD) and catalase (CAT) enzymatic functions, breaking down H_2_O_2_ into hydroxyl radicals (˙OH) and oxygen, administering chemotherapy to alleviate hypoxia. Additionally, MSCs@PEG function as GSH oxidase (GSHOD), depleting intracellular GSH and disrupting the redox balance, thus enhancing oxidative stress. Furthermore, MSCs@PEG showcase unique biodegradability, enabling elimination through urine and feces within a 14-day period. The synergistic combination of PTT, PDT, and chemotherapy equips MSCs@PEG with remarkable anticancer efficacy and immune activation.^74^

In a monolayer of MoS_2_, the atomic arrangement involves covalent bonding in the S–Mo–S structure, resembling a sandwich-like formation. Meanwhile, neighboring MoS_2_ layers are connected through less robust van der Waals interactions. As a result, it is straightforward to extract MoS_2_ nanosheets (NSs) from MoS_2_ blocks. Various techniques have been developed for the production of MoS_2_ NSs, encompassing mechanical, liquid, chemical, and electrochemical exfoliation, alongside methods such as chemical vapor deposition, high-temperature decomposition, and solvent thermal approaches, among others. Building upon single-layer MoS_2_ NSs, enhancing their diagnostic and therapeutic capabilities involves introducing imaging contrast agents or therapeutic drugs through synergistic interactions and physical adsorption. Cai *et al.* introduced a straightforward one-pot method that achieves the simultaneous exfoliation and *in situ* functionalization of single-layer MoS_2_ nanosheets (Fig. 3f).^75^ This approach involves utilizing gadolinium oxide nanoparticles templated by bovine serum albumin (BSA-Gd_2_O_3_) as both the exfoliating agent and T1 contrast agent for magnetic resonance imaging (MRI). Subsequently, hyaluronic acid (HA) was additionally incorporated to facilitate the targeting of cancer cells characterized by CD44 overexpression. Moreover, the nanomaterial was loaded with gambogic acid (GA), a naturally occurring inhibitor of heat shock protein 90 and an antitumor drug. This integration aimed to mitigate the thermal resistance exhibited by tumor cells, enabling successful PTT within a gentle temperature range of 43–45 °C. Through a combination of *in vivo* and *in vitro* experiments, the study verified the favorable biocompatibility of the GA/MoS_2_/BSA-Gd_2_O_3_-HA nanocomposite. This nanocomposite exhibits promise for applications combining low-temperature PTT with chemotherapy guided by MRI.

### 3.2 PDT and PTT of WS_2_

**Fig. 4:**
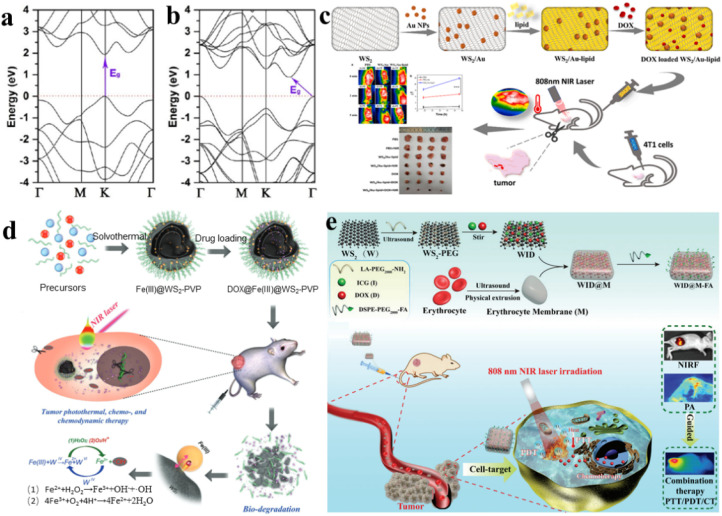
(a) Electronic structure of monolayer WS_2_.^64^ (b) Bulk WS_2_ electronic band structure.^64^ (c) Synthesis schematic of WS_2_/Au-lipid and its utilization in combined photo-chemotherapy for anti-tumor applications.^81^ (d) Schematic representation of solvothermal synthesis, drug loading, tumor PTT, chemo- and nanocatalytic chemodynamic therapy (CDT).^82^ (e) Schematic representation of the preparation of WID@M-FA nanosheets and the theranostic process for imaging-guided chemo/photothermal therapy.^83^

Recently, experimental observations have confirmed the presence of photoluminescence (PL) signals in monolayered WS_2_.^76^ Additionally, our monolayered WS_2_ films also exhibit photoluminescent activity. In the bulk phase of WS_2_, there exists an approximately 1.4 eV indirect electronic band gap, along with a direct electronic band gap measuring 2.01 eV.^77^ In contrast, single-layered WS_2_ exhibit a direct band gap of around 1.9 eV, which closely aligns with the predictions from density functional theory within the local density approximation (DFT-LDA) calculations.^78^Fig. 4a and b present the electronic band structures computed for both monolayer and bulk WS_2_.^64^ It is clear that WS_2_ exhibits NIR absorption.

As we know, the integration for combined chemotherapy and photothermal therapy for cancer cell destruction has emerged as a significant research focus, addressing the limitations of conventional cancer treatments.^79^ Au NPs are known for their good biocompatibility, ease of surface modification, lack of toxicity, and straightforward preparation. Incorporating WS_2_ with gold nanoparticles yields a composite exhibiting remarkable near-infrared absorption and photothermal conversion capabilities.^80^ WS_2_ nanosheets serve as the core material, onto which gold nanoparticles are incorporated, resulting in a tungsten disulfide surface doped with gold (WS_2_/Au), which is later coated with lipids to create WS_2_/Au-lipids (Fig. 4c).^81^ This functional nanocomposite exhibits heightened biocompatibility and stability under physiological conditions. Notably, WS_2_/Au-lipid demonstrates excellent photothermal conversion efficiency, as validated through both *in vitro* and *in vivo* photothermal experiments. Moreover, this WS_2_/Au-lipid platform carrying the chemotherapy agent DOX displays a dual-responsive release profile, triggered by both pH and NIR stimuli. *In vitro* experiments involving combined photothermal and chemotherapy treatments reveal a significantly lower cell survival rate in the combined treatment group compared to the exclusive treatment cohort. *In vivo* investigations of PTP indicate improved efficacy post-modification, with enhanced tumor targeting and heightened drug concentration within cancer cells. Importantly, the *in vivo* study affirms that the combined treatment cohort exhibits more pronounced therapeutic effects on tumors; the impact was greater than in the exclusive treatment cohort, while demonstrating no observable generalized toxicity.

More remarkably, the generation of Fe^2+^ and the release of DOX are additionally propelled by the elevated levels of H_2_O_2_ and the slightly acidic conditions in the tumor microenvironment. This acceleration occurs because H^+^ and H_2_O_2_ can expedite the conversion of Fe^2+^ through oxidation. The continuously produced Fe^2+^ triggers a swift Fenton reaction with the endogenous H_2_O_2_ within tumor cells, generating a substantial amount of highly toxic hydroxyl radicals for nanocatalytic tumor treatment. Combined with outstanding photothermal transformation capabilities, the DOX@Fe(iii)@WS_2_-PVP nanocapsules effectively trigger intrinsic redox reactions and amplify photothermal therapy within the tumor microenvironment, thereby leading to a synergistic chemo and nanocatalytic therapy outcome (Fig. 4d).^82^ Within these nanocapsules, an oxidation–reduction reaction takes place involving WS_2_ and Fe(iii) species, resulting in the formation of WO_4_^2−^ and Fe^2+^. The generated Fe^2+^ can undergo oxidation to Fe^3+^, which subsequently reacts with Fe(iii)@WS_2_-PVP to perpetually generate WO_4_^2−^ and Fe^2+^. This recurring endogenous redox reaction significantly enhances the biodegradation and release of DOX from DOX@Fe(iii)@WS_2_-PVP. Remarkably, the production of Fe^2+^ and the release of DOX are further accelerated by the overexpression of H_2_O_2_ and the mildly acidic tumor microenvironment. This is due to the acceleration of Fe^2+^ oxidation by H_2_O_2_ and H^+^, which subsequently catalyzes a rapid Fenton reaction with the intrinsic H_2_O_2_ within tumor cells. This reaction yields an abundance of hydroxyl radicals, thus serving as a potent nanocatalytic strategy for cancer treatment. Coupled with a strong ability for photothermal conversion, the DOX@Fe(iii)@WS_2_-PVP nanocapsules effectively fulfill their objectives, both through an inherent oxidation–reduction reaction and an externally enhanced photothermal treatment for tumors within the tumor microenvironment. This comprehensive approach encompasses chemotherapy and nanocatalytic therapy outcomes.

The substantial adverse reactions associated with pharmaceutical drugs and the challenge of drug resistance across multiple agents pose significant hurdles to effective tumor therapy. Consequently, a novel approach involving the integration of chemotherapy and photothermal therapy (CT/PT) has gained attention, offering a solution with reduced drug dosages. The progress in multifunctional advancements in drug delivery technologies, capable of enhancing immune function evasion and improving drug concentration in targeted tumor tissues, is currently in its nascent stages. It was reported that nanosheets of tungsten disulfide (WS_2_) modified with polyethylene glycol (PEG) (referred to as WID) were employed as a nanocarrier framework for incorporating DOX and introducing the near-infrared indocyanine green (ICG) as a fluorescence probe (Fig. 4e).^83^

Through surface modification with a red blood cell membrane (M) and the specific targeting of folic acid (FA) molecules, an innovative biomimetic platform, designated as WID@M-FA NPs, was engineered. This system exhibited elevated biocompatibility, an extended circulation period (a 3.6-fold increase compared to WID NPs), along with remarkable NIR photothermal functionality, all contributing to a targeted approach for the treatment of cervical cancer. *In vitro* assessments demonstrated that the photothermal effects generated by ICG under laser irradiation not only augmented cellular drug uptake but also enhanced the efficiency of tumor cell destruction. Furthermore, the targeted delivery of DOX to cervical cancer tissues and the synergistic chemo/photothermal therapy resulted in tumor eradication of over 95%, without inducing side effects in normal tissues during *in vivo* experiments.

### 3.3 PDT and PTT of FeS_2_

**Fig. 5:**
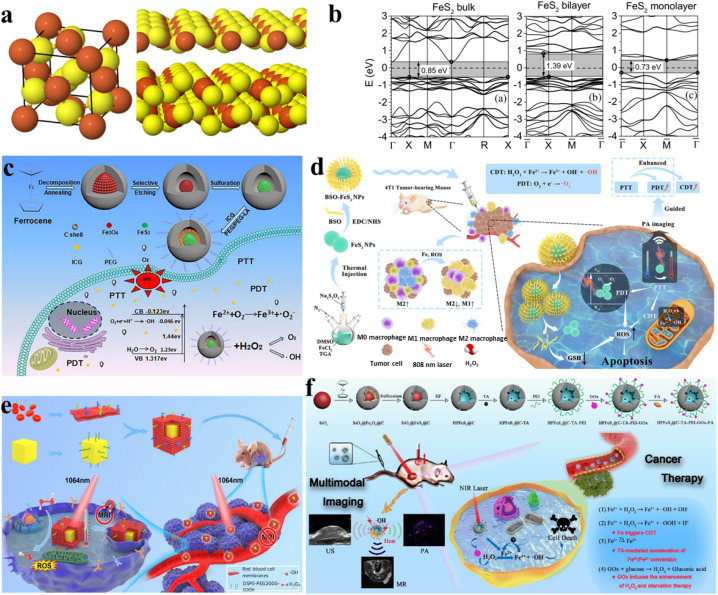
(a) Models of FeS_2_ in bulk, monolayer, and bilayer forms, with S atoms in yellow and Fe atoms shown in brown.^84^ (b) Band structures and densities of states (DOS) for bulk, bilayer, and monolayer FeS_2_, with shaded regions indicating the band gaps.^84^ (c) Combining PTT, PDT, and multimode imaging into a single nanoplatform.^86^ (d) Illustration of PAI-guided PTT, CDT and PDT mediated by BSO-FeS_2_.^87^ (e) Schematic depicting the *in vivo* fabrication and anti-tumor impact of FeS_2_@RBCs. The RBC coating results in extended blood circulation, enhancing tumor accumulation.^88^ (f) Preparation procedure and therapeutic mechanism of HPFeS_2_@C-TA-PEI-GOx-FA.^89^

The volume and surface electronic structure of FeS_2_ are highly significant, especially in studying the reduction of thickness to a single layer through DFT calculations as shown in the spherical model of bulk FeS_2_ as well as its monolayer, and bilayer forms (Fig. 5a).^84^Fig. 5b illustrates the band structures near the Fermi level and its respective density of states (DOS) for bilayer FeS_2_ (1.39 eV), bulk FeS_2_ (0.85 eV), and monolayer FeS_2_ (100) (0.73 eV). In the band structure of the bulk material, the valence band appears modestly narrow and is clearly distinguishable from the bottom bands.^84^ Due to the wide light absorption, FeS_2_ is extensively employed as a highly effective photocatalyst for oxidizing pollutants including methyl orange, bisphenol A, rhodamine B, and ciprofloxacin. This efficacy is attributed to the generation of ROS by FeS_2_. Due to its ability to generate ROS, FeS_2_ is also considered as a potential agent for PDT. It is noted that the generation of ROS relies heavily on the concentration of O_2_. The microenvironment within tumors typically experiences higher hypoxia (lower oxygen levels) compared to normal tissues, which can impact the effectiveness of PDT. To address this issue and enhance the O_2_ concentration, many reports suggest the utilization of intracellular H_2_O_2_.^85^ This strategy aims to increase the availability of oxygen for the generation of ROS, ultimately improving the efficacy of FeS_2_ in photodynamic therapy for treating tumors. Li *et al.* synthesized FeS_2_@C yolk–shell nanomaterials integrating PTT and PDT on a single platform (Fig. 5c) Fe_3_O_4_@C was synthesized through a straightforward single-step hydrothermal technique, followed by fractional etching of the Fe_3_O_4_ core to create the yolk–shell structure of Fe_3_O_4_@C.^86^ Subsequently, FeS_2_@C, approximately 200 nm in size, was obtained through straightforward sulfuration. This nanomaterial exhibited efficient NIR harvesting and remarkable photothermal transformation due to its unique void architecture and narrow band gap (1.52 eV). As anticipated, under NIR illumination, FeS_2_@C yolk–shell nanomaterials demonstrated the generation of reactive ROS. Experiments additionally revealed that ˙O_2_^−^ and ˙OH were the primary functioning species. The mechanism of ROS generation was thoroughly investigated, revealing that dissolved O_2_ and photo-excited electrons played a pivotal role. Furthermore, FeS_2_@C could oxidize water under NIR light, alleviating oxygen deficiency in cancer cells and enhancing photodynamic therapy efficacy. This was attributed to the sufficient VB potential of FeS_2_. Additionally, Fe ions participating in the Fenton reaction facilitated intracellular H_2_O_2_ degradation by FeS_2_, generating O_2_ and ˙OH to support photodynamic therapy. Moreover, photosensitizer ICG was loaded into the material to enhance PDT and PTT effects. Incorporating MRI and leveraging the synergistic effects of PDT and PTT, FeS_2_@C-ICG-PEG emerges as a promising nanotheranostic agent for cancer treatment.

Xiao *et al.* synthesized ultrasmall FeS_2_ nanoparticles, which were modified by utilizing BSO, resulting in BSO-FeS_2_ NPs. This modification aimed to enhance combined chemo-photodynamic therapy (CDT/PDT) effectiveness under 808 nm laser irradiation for photothermal enhancement (Fig. 5d).^87^ The ultrasmall FeS_2_ NPs, characterized by their extensive surface area, not only expanded the contact surface with reactants such as H_2_O_2_ and O_2_ but also amplified the incident light concentration, leading to an increased production of reactive ROS. Additionally, these NPs served as photoacoustic imaging (PAI) contrast agents. Moreover, the elevated intracellular Fe and ROS levels induced by BSO-FeS_2_ NPs can induce the repolarization of tumor-associated macrophages (TAMs), shifting them from the immunosuppressive M2 phenotype to the tumoricidal M1 phenotype. This process involves a transformation in cellular behavior. This change notably improved the effectiveness of tumor immunotherapy. As a result, BSO-FeS_2_ NPs emerged as promising “all-in-one” theranostic agents for cancer treatment involving PAI-mediated PTT/CDT/PDT.^86^ Similarly, She *et al.* introduced a rational design involving red blood FeS_2_@RBCs for improved MRI-guided applications hyperthermia-enhanced photothermal therapy (HPTT) and CDT in synergistic cancer treatment (depicted in Fig. 5e). First, FeS_2_@RBCs demonstrated robust adsorption, enhanced blood circulation and absorption in the NIR-II window, and improved tumor accumulation for effective cancer HPTT. Additionally, the hyperthermia-induced FeS_2_@RBCs enhanced the CDT effect, leading a synchronized synergistic therapy combining HPTT and CDT. Moreover, enhanced MRI in the tumor microenvironment (TME) facilitated the observation of nanoparticle accumulation in the tumor area, aiding in pre-treatment guidance. The results from *in vitro* and *in vivo* experiments demonstrated the significant therapeutic efficacy of FeS_2_@RBCs at an FDA-approved laser intensity density of 1.0 W cm^−2^ for 1064 nm. This advancement could potentially pave the way for the clinical application of the synergistic CDT and HPTT.^88^

It is noted that the effectiveness of tumor treatment is constrained by the effectiveness of chemical reactions and heavily depends on catalysts. Wu *et al.* addressed this by developing and utilizing the HPFeS_2_@C nanocatalyst for a triple-enhanced CDT (Fig. 5f).^89^ Tannic acid encapsulation within HPFeS_2_@C aimed to convert Fe^3+^ to Fe^2+^, enhancing catalytic activity and accelerating the Fenton reaction. Subsequently, in the nanocatalysts, glucose oxidase (GOx) utilized glucose from the tumor microenvironment to produce H_2_O_2_ on-site or at the original location, thus enhancing Fenton reaction efficiency. This glucose consumption also induced a starvation effect, contributing to starvation therapy for cancer. Furthermore, the photothermal characteristics of HPFeS_2_@C induced heat, accelerating the Fenton process and facilitating synergistic photothermal therapy, starvation, and CDT.

### 3.4 PDT and PTT of Bi_2_S_3_

**Fig. 6:**
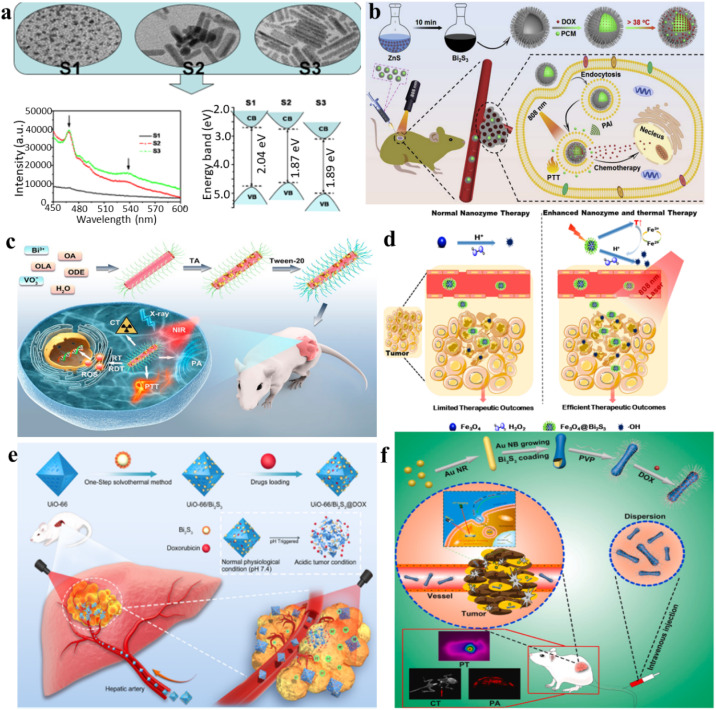
(a) Transmission electron microscopy (TEM) images and photoluminescence (PL) patterns of Bi_2_S_3_ nanocrystals synthesized at 100 °C for varying reaction times.^90^ (b) A diagram illustrating the thermosensitive urchin-like structure of Bi_2_S_3_ hollow microspheres used as carriers for DOX, enabling photoacoustic imaging and combining photothermal–chemo therapy for tumors.^91^ (c) Schematic overview depicting the synthesis and applications of Tween-20-modified BiVO_4_@Bi_2_S_3_ HNRs for multimodal CT/PA imaging and synergistic therapy involving RT/RDT/PTT.^92^ (d) Schematic diagram of the tumor-specific sequential treatment mechanism of Fe_3_O_4_@Bi_2_S_3_ under photothermal and photothermal-enhanced nanozyme catalysis at 808 nm.^93^ (e) A one-step solvothermal approach was employed for the synthesis of multifunctional nanoparticles (UiO-66/Bi_2_S_3_@DOX), enabling the concurrent realization of photothermal effects and pH-responsive DOX release.^94^ (f) Au@Bi_2_S_3_-PVP NBs loaded with DOX were developed for the combined application of PT/PA/CT imaging and synergistic chemo/PT therapy against liver cancer.^95^

Various shapes and sizes of Bi_2_S_3_ nanocrystals are obtained through a hot injection method. Colloidal entities shift between nanodot and nanorod structures, exhibiting dimensions ranging from 3–4 nm to 40–50 nm. It is clear that a blue shift is evident in the band gap at energy levels of 2.04, 1.87, and 1.89 eV from nanodots to nanorods, respectively. Notably, the crystallinity and morphology, as well as the photoluminescence emission are significantly affected by the nanocrystals, decreasing for nanodots and increasing for nanorods with higher aspect ratios (Fig. 6a).^90^ The shape and size of Bi_2_S_3_ may have an important impact on its light response. On the other hand, the narrow bandgap of Bi_2_S_3_ indicates it NIR absorption capability, which has been utilized for PTT and PDT.

For example, Zhao *et al.* developed a simple and rapid synthetic method to create large-scale hollow microspheres of Bi_2_S_3_ exhibiting rod-based urchin-like nanostructures, denoted as U-BSHM. It was synthesized through a sacrificial template approach, with ZnS composite microspheres serving as the templates (Fig. 6b).^91^ An investigation into the growth mechanism of U-BSHM was undertaken by adjusting the amount of Bi source and observing the morphological evolution of intermediate products. Doxorubicin hydrochloride was effortlessly incorporated into the inner region of U-BSHM, along with the 1-tetradecanol phase change material (PCM), acting as a “gatekeeper” to regulate the release of DOX upon exposure to NIR light-induced temperature increase. The photothermal influence exerted by U-BSHM triggered the PCM phase conversion from solid to liquid due to localized temperature elevation. This enabled precise pulsed drug release from within the hollow spaces, a mechanism that was thoroughly examined to emphasize its advantages. Ultimately, the rod-based U-BSHM emerged as an ideal nanotheranostic agent, (DOX + PCM)@Bi_2_S_3_, for tumor treatments, offering photoacoustic imaging and photothermal-chemo therapy capabilities.^92^ Wang *et al.* have presented the creation and synthesis of BiVO_4_@Bi_2_S_3_ heterojunction nanorods (HNRs) modified with Tween-20 for a synergistic therapy approach involving multimodal imaging involving computed tomography (CT) and photoacoustic (PA), along with therapies combining radiotherapy (RT), radiodynamic therapy (RDT), and PTT. Utilizing the heightened X-ray attenuation coefficient of bismuth (Bi), the HNRs composed of BiVO_4_@Bi_2_S_3_ exhibit notable capabilities in CT imaging and enhance radiation effects during radiotherapy (RT). Simultaneously, the robust NIR absorption exhibited by Bi_2_S_3_ imparts remarkable photoacoustic (PA) imaging and photothermal conversion capabilities to the BiVO_4_@Bi_2_S_3_ HNRs (Fig. 6c).^92^

Nanomaterials with intrinsic peroxidase-like activities have been explored as synthetic enzymatic agents for cancer therapy, catalyzing substrate oxidation using peroxides. However, the reliance on hydrogen peroxide and pH in current peroxidase catalytic oxidation treatments limits their efficacy within the tumor microenvironment. Researchers led by Zhao *et al.* have introduced an innovative approach involving the construction of complex virus-like nanocatalysts termed Fe_3_O_4_@Bi_2_S_3_ (referred to as F-BS NCs) (Fig. 6d).^93^ These nanocatalysts combine the peroxidase properties of conventional Fe_3_O_4_ (magnetic nanoparticles) with the attributes of the narrow band gap semiconductor, Bi_2_S_3_ (BS), to enhance enzymatic activity by utilizing effective external photothermal stimuli and the constrained intratumoral peroxide levels. Within in this formulation, the combined F-BS NCs induce apoptosis in cancer cells through a mild photothermal treatment, followed by sequential photothermal-stimulated catalysis of H_2_O_2_ into ˙OH radicals upon exposure to 808 nm laser irradiation. This successful integration achieves a remarkable synergistic anticancer effect, addressing the limitations of current therapeutic strategies.

Transcatheter arterial transarterial chemoembolization (TACE) stands as the preferred therapeutic approach for patients with intermediate-stage hepatocellular carcinoma (HCC). Nevertheless, it struggles to eliminate all cancer cells and lacks specificity, causing damage to healthy liver cells. In recent developments, the integration of nano-delivery and PPT systems has been utilized to augment the effectiveness of TACE. Nevertheless, these strategies mainly achieve single functions and involve complex synthesis procedures. Liu *et al.* utilized a facile one-pot solvothermal method to fabricate multifunctional nanoparticles (UiO-66/Bi_2_S_3_@DOX) to induce photothermal effects and initiate low-pH-dependent release of DOX simultaneously (Fig. 6e).^94^ UiO-66/Bi_2_S_3_ exhibits responsive release behavior to pH changes and displays exceptional photothermal effects, as demonstrated through various *in vitro* and *in vivo* studies. The confirmed biocompatibility is further supported by cell toxicity and blood compatibility assessments. In a rat N1S1 liver tumor model, the combined application of TACE and PTT results in significant suppression of tumor growth, as evidenced by extensive necrosis upon histopathological examination.

For liver cancer, it was reported that the amalgamation of gold nanorods with a bismuth sulfide (Bi_2_S_3_) film resulted in the formation of Au@Bi_2_S_3_ nano-bones (NBs), which exhibited outstanding photoacoustic (PA) imaging capabilities, ultrahigh photothermal (PT) conversion efficiency, and high-performance computed tomography (CT); this combination showcases remarkable multifunctionality (Fig. 6f).^95^ The Au@Bi_2_S_3_ nanobeads exhibit significant potential as a nanotheranostic agent for PT/PA/CT imaging. Following this, the successful loading of the anticancer drug DOX onto the poly(*N*-vinylpyrrolidone)-modified Au@Bi_2_S_3_ nanobeads (Au@Bi_2_S_3_-PVP NBs) results in a favorable pH-sensitive release profile. This reveals the significant capability of Au@Bi_2_S_3_-PVP nanobeads (NBs) in chemotherapy as they function as drug carriers, facilitating the delivery of DOX into cancer cells. The results from both *in vitro* and *in vivo* studies affirm that Au@Bi_2_S_3_-PVP nanobeads (NBs) exhibit numerous advantageous attributes for cancer therapy. These comprise efficient accumulation, precise tumor targeting, remarkably low toxicity, excellent biocompatibility, and a high capacity for drug loading. Au@Bi_2_S_3_-PVP NB-mediated PTT achieved highly efficient ablation of human liver cancer cells (HepG2). Functioning as both a contrast enhancement probe and therapeutic agent, Au@Bi_2_S_3_-PVP nanobeads (NBs) demonstrated exceptional near-infrared-triggered multi-modal PT/PA/CT imaging-guided PTT. Furthermore, they effectively suppressed the growth of HepG2 liver cancer cells through synergistic chemo/PT therapy. Steady-state and transient-state fluorescence spectroscopy elucidate the pathways of cross-relaxation and the mechanism of energy migration.^34^ Capabilities in photothermal conversion and production of ROS were investigated *via* upconversion and downconversion luminescence modes. Both *in vitro* and *in vivo* antitumor studies under 808 nm laser irradiation confirm the advantageous characteristics of the core–shell structure of NPs. The cancer-cell-specific cytotoxicity of the synthesized UCNPs@AgBiS_2_ core–shell NPs ensures enhanced therapeutic efficacy, as expected.

### 3.5 PDT and PTT of other transition metal sulfides

ReS_2_ is composed of three atomic layers arranged in an S–Re–S configuration, where Re (rhenium) and S (sulfur) atoms are connected through covalent bonds. Like other prominent 2D materials, van der Waals forces weakly couple the adjacent layers in ReS_2_, resulting in the formation of bulk crystals. This layering arrangement is a common feature in many 2D materials and contributes to their unique properties. Unlike some other TMDs, ReS_2_ demonstrates layer-independent electrical, optical, and vibrational properties.^95,96^ While other well-studied TMDs have molecular structures labeled as 1H, 2H, 3R, or 1T phases, the unit cell of ReS_2_ is derived from hexagonal symmetry, transitioning towards a distorted 1T structure.^97^ In this structure, Re atoms form parallelograms consisting of four Re atoms. This arrangement introduces built-in planar anisotropy, offering versatile possibilities for constructing composite heterostructures. This distinctive structure imparts in-plane anisotropy to ReS_2_, leading to variations in its fundamental physical properties along different directions within the plane. ReS_2_ is mechanically flexible and interacts strongly with incident light. This characteristic enhances photon absorption and promotes the generation of electron–hole pairs. Thus, ReS_2_ stands out for maintaining consistent physicochemical properties between its 2D and 3D forms, layer-independent optoelectronic properties, a lack of shift in the optical absorption range, mechanical flexibility, strong interaction with light, and the potential for designing functional heterostructures.

**Fig. 7:**
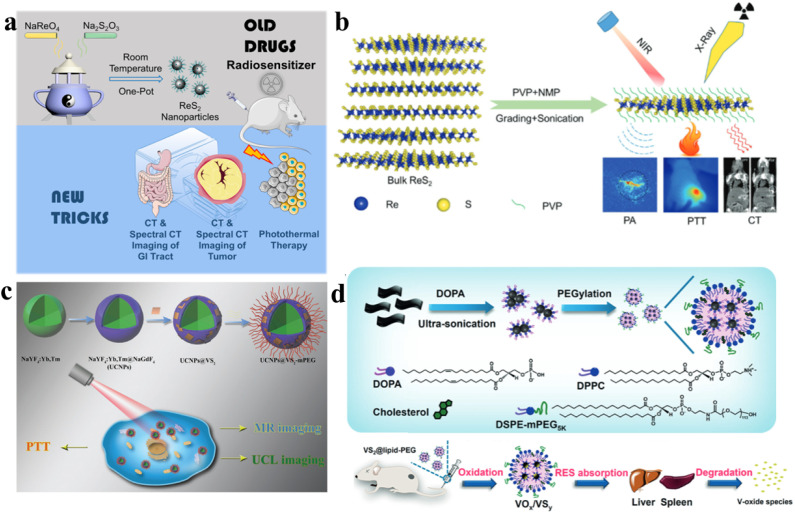
(a) The strategy of “Teaching Old Drugs New Tricks”, a schematic showcasing the application of ReS_2_ nanoparticles for both GI tract spectral CT imaging and tumor theranostics.^98^ (b) The exfoliation process of PVP-capped ReS_2_ nanosheets; this scheme guides bimodal PA and CT imaging for photothermal therapy.^99^ (c) With the combined PTT treatment, the *in vivo* processes of oxidation, degradation, and clearance for VS_2_@lipid-PEG nanoparticles occur.^103^ (d) The manufacturing procedure for VS_2_@lipid-PEG. Visual representation elucidating the *in vivo* oxidation, degradation, and clearance processes of VS_2_@lipid-PEG nanoparticles.^104^

Moreover, due to its high atomic number (*Z* = 75), the ReS_2_ nano-agent is expected to exhibit strong X-ray absorption ability. This property positions it as having great potential for spectral computed tomography (CT) imaging. Spectral CT imaging involves acquiring and analyzing X-ray data at multiple energy levels, providing enhanced contrast and improved tissue characterization. The strong X-ray absorption ability of ReS_2_ makes it a promising candidate for advancing the capabilities of spectral CT imaging in medical diagnostics. Wang *et al.* presented the fabrication of sub-10 nm-sized rhenium disulfide nanoparticles, functioning as a sensitizer in clinical radiotherapy and as a biologically safe contrast agent for spectral CT (Fig. 7a).^98^ By applying innovative techniques to a well-established drug, these nanoparticles were employed for *in vivo* imaging of the gastrointestinal tract and diagnostic therapy for tumors. The synthesis of ReS_2_ nanoparticles was achieved through a straightforward single-step procedure at ambient temperature, demonstrating not only a size below 10 nm with outstanding monodispersity, but also remarkable X-ray attenuation capability and solubility in water. Moreover, these nanoparticles exhibited exceptional spectroscopic CT imaging performance and unquestionable safety for clinical therapeutic applications. Furthermore, owing to their potent NIR absorption, the ReS_2_ nanoparticles showcased promising photothermal capabilities. This innovative nano-agent not only provided prominent contrast enhancement in *in vivo* gastrointestinal tract spectral CT imaging but also facilitated effective CT imaging-guided photothermal therapy for tumors. Miao *et al.* introduced a scalable and continuous synthesis of colloidal ReS_2_ nanosheets using a liquid exfoliation method assisted by probes (Fig. 7b).^99^ This technique was investigated as a theragnostic agent for the diagnosis of cancer and treatment. Owing to the high atomic number of rhenium (Re) and its remarkable photoacoustic effect, the PVP-capped ReS_2_ nanosheets obtained were evaluated as bimodal contrast agents suitable for both photoacoustic imaging and computed tomography. Additionally, leveraging robust near-infrared absorption and an exceptionally high photothermal conversion efficiency of 79.2%, the ReS_2_ nanosheets were found to have therapeutic potential, achieving a complete elimination rate of up to 100% for the photothermal ablation of tumors. Crucially, the ReS_2_ nanosheets exhibited minimal toxicity, as confirmed through cytotoxicity assays, serum biochemistry evaluations, and histological analysis. This study underscores the potential of ReS_2_ nanosheets as a multifunctional monocomponent theragnostic nanoplatform, serving dual purposes in both bioimaging and anti-tumor therapy.

From the perspective of the crystal structure, a hexagonal structure exhibiting the P3̄ml space group configuration is demonstrated by VS_2_. In alignment with the (001) plane, the VS_2_ lattice is constructed with sandwiched S–V–S monolayers, where metal/V layers are positioned between two S layers. These monolayers stack to form a stratified arrangement; the structure exhibits an interlayer separation of 5.76 Å, maintained by feeble van der Waals interactions.^100^ In H-VS_2_ and T-VS_2_ layers, in the trigonal prism and octahedron, V atoms are situated at the central positions, respectively. Both structures display a 2D hexagonal lattice, characterized by periodicity along the *a*/*b* directions that run parallel to the VS_2_ plane.^101^ Theoretical studies show that VS_2_ layers, including both H and T phases, inherently display metallic or conductive properties. This is evidenced by the elevated local density of states (DOS) crossing. The Fermi level, coupled with the absence of a zero bandgap, indicates a promising prospect for high microscopic 2D conductivity.^102^ In addition, VS_2_ has primarily been employed in enabling imaging, drug delivery, and phototherapy applications. In nanocomposites utilizing up-conversion nanoparticles (UCNPs) and photothermal agents, the usual practice involves separate pre-synthesis of the two types of nanoparticles. Subsequently, they are assembled together through physical or chemical means to create the nanocomposites. The process of synthesis involving the amalgamation of these components is frequently complex and time-consuming. Hence, there is a substantial need to investigate a gentle and streamlined approach. Wang *et al.* developed a novel and straightforward approach to create a heterogeneous combination of luminescent UCNPs with vanadium disulfide (VS_2_) grown on their surface (Fig. 7c).^103^ The growth of VS_2_ directly onto UCNPs yielded oil-soluble nanocomposites, termed UCNPs@VS_2_. Subsequently, the introduction of polyethylene glycol (mPEG) functionalized the nanocomposites, resulting in an integrated nanostructure named UCNPs@VS_2_-mPEG. This modification improved water solubility, and the resulting composite had an estimated size of around 25 nm, making it suitable for *in vitro* photothermal therapy. Crucially, cytotoxicity tests confirmed the biocompatibility of the final nanostructure. Leveraging the outstanding photothermal properties of VS_2_ combined with the distinctive imaging capabilities of UCNPs, effectively performing photothermal therapy on HeLa cells, this nanostructure demonstrated successful applications in *in vitro* upconversion luminescence imaging and magnetic resonance imaging. Hence, this research showcases a straightforward yet potent method for the cultivation of VS_2_ on the surface of UCNPs, offering an efficient approach to create a nanoscale combined structure for treatment and dual-model bioimaging. After lipid modification, VS_2_ nanosheets can undergo a transformation into ultra-small VS_2_ nanodots encapsulated within polyethylene glycol (PEG)-modified lipid micelles. Liu *et al.* obtained VS_2_@lipid-PEG nanoparticles with a photothermal conversion efficiency of 31.5%. Owing to paramagnetism, strong NIR absorbance, and chelator-free labeling with ^99m^Tc^4+^, the VS_2_@lipid-PEG nanoparticles can be used for tri-modal imaging (T1-weighted magnetic resonance, photoacoustic, and single-photon emission computed tomography) and guide photothermal ablation of tumors (Fig. 7d).^104^ This innovative approach not only represents the initial demonstration of *in vivo* MR imaging using VS_2_ but also achieves highly efficient multimodal imaging. Guided by imaging, the gradual degradation of VS_2_ into molecular species is crucial for photothermal tumor ablation, allowing for efficient clearance of VS_2_@lipid-PEG nanoparticles without inducing toxicity at the tested dosage level in mice. Notably, these VS_2_@lipid-PEG nanoparticles exhibit exceptional stability across various physiological solutions. The exceptional photothermal conversion efficiency and robust photostability of VS_2_@lipid-PEG establish it as an outstanding PTT nano-agent.

## 4. Sonodynamic therapy of transition metal sulfides

### 4.1 Possible SDT mechanisms

Sonosensitizers designed for SDT fall into two categories: (1) semiconductor-based sonosensitizers: these materials, upon absorbing energy, undergo a series of reactions where electrons and holes are separated, leading to the generation of reactive ROS to achieve the SDT effect. (2) Cavitation-based sonosensitizers: sonosensitizers are substances that enhance the effects of ultrasound-based therapeutic procedures. In this context, the sonosensitizers are associated with cavitation, a phenomenon where the rapid formation and collapse of bubbles occur in a liquid exposed to ultrasound. The rupture of cavitation bubbles leads to changes in the microenvironment. This could include alterations in temperature, pressure, and the release of reactive species. These changes in the microenvironment can be harnessed for various applications, particularly in the context of therapeutic interventions. Sonosensitizers can absorb the energy released during the collapse of cavitation bubbles. This energy absorption can result in the generation of ROS and other cytotoxic effects, which are utilized for targeted therapy, particularly in cancer treatment.^105^

**Fig. 8:**
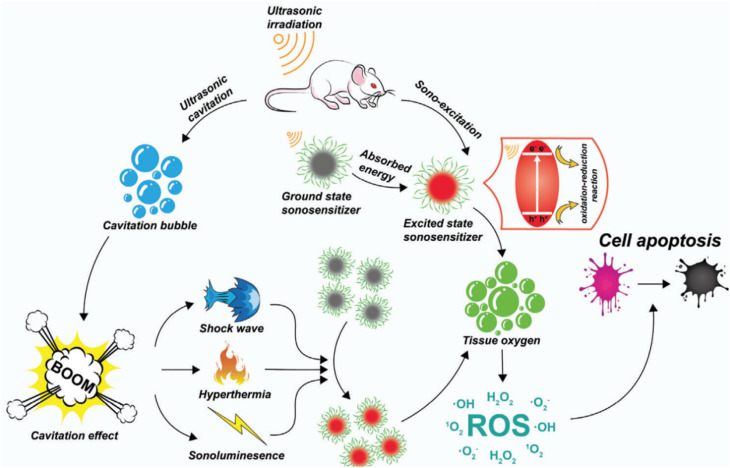
Different possible SDT mechanisms.^106^

The mechanism resembles that of a photosensitizer absorbing light and subsequently releasing reactive ROS. In the case of organic sonosensitizers, those within the tumor absorb energy under ultrasound, prompting electrons to transition to the excited state from the ground state. Upon returning to the ground state, the liberated energy interacts with oxygen or water, resulting in the generation of ROS. In the case of inorganic sonosensitizers, energy absorption during ultrasound exposure generates separated holes (h^+^) and electrons (e^−^). Following that, the electrons and holes engage in reactions with water and oxygen, respectively, leading to ROS formation (Fig. 8).^106^ Furthermore, ultrasonic cavitation plays a pivotal role. During cavitation, bubbles nucleate, expand, and burst due to the ultrasound. The energy released during bubble bursting is distributed in three ways. Firstly, it alters the local pressure, generating shock waves that transmit pressure to the surrounding environment (up to 81 MPa). The alteration in pressure influences the charge distribution of piezoelectric materials, leading to internal electric fields that interact with oxygen within the tumor microenvironment, resulting in the generation of ROS. Secondly, the occurrence of sonoluminescence occurs as the bubbles burst, emitting light of specific wavelengths. Sonosensitizers absorb this emitted light and then undergo reactions with oxygen and water, leading to the production of ROS. Lastly, cavitation-driven energy raises the local temperature significantly (up to 10 000 K), leading to water splitting and producing ˙OH, which reacts with internal substances to generate ROS. Although the exact mechanism remains somewhat unclear, ROS production stands as the primary contributor to ultrasound-induced cell death.^107,108^

### 4.2 SDT of MoS_2_

**Fig. 9:**
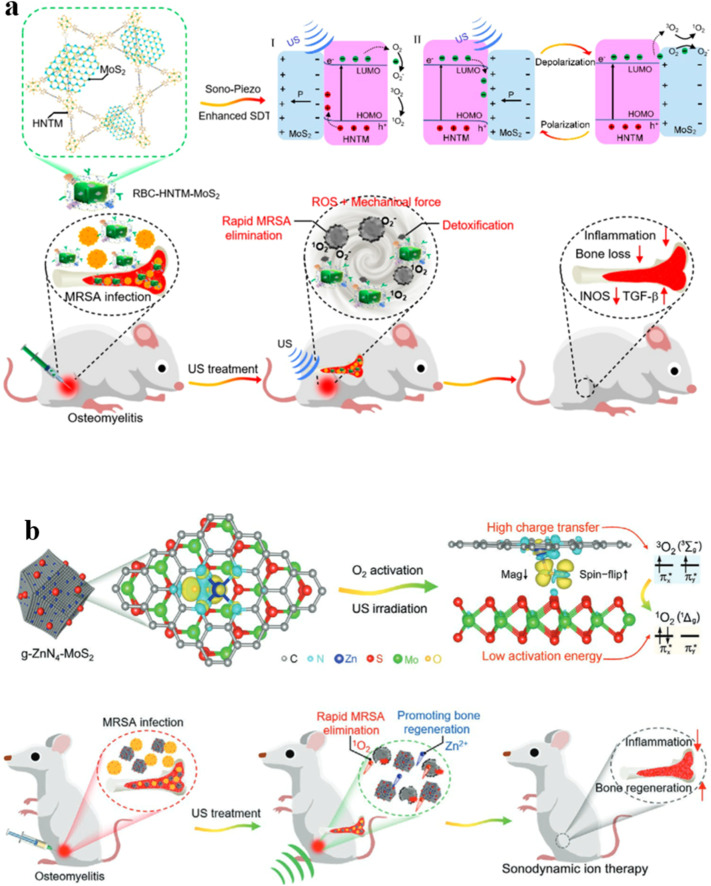
(a) Mechanism of sonocatalysis and the efficient treatment of osteomyelitis through sonodynamic therapy using the HNTM-MoS_2_ heterointerface.^111^ (b) Sonocatalytic mechanism for effectively treating osteomyelitis with g-ZnN_4_-MoS_2_ in efficient sonodynamic therapy.^112^

Effectively treating osteomyelitis remains a substantial challenge in the realm of orthopedics.^109^ Combining long-term systemic antibiotic therapy and surgical debridement is often necessary for managing refractory bone infections clinically. There is an urgent need to develop a strategy that is antibiotic-free, non-invasive, and rapid for eradicating osteomyelitis.^110^ SDT has been proven to be an efficient strategy to treat osteocarcinoma. Feng *et al.* proposed a novel approach to create a piezoelectric-enhanced sonosensitizer, which consists of a red blood cell (RBC) membrane, MoS_2_ nanosheets, and a porphyrin-based hollow metal–organic framework (HNTM) (Fig. 9a).^111^ The research revealed that the piezoelectric polarization induced by ultrasound (US) in MoS_2_ enhances the charge transfer at the HNTM–MoS_2_ heterointerface, thereby increasing the generation of ROS. Additionally, MoS_2_ contributes to the asymmetric shaping of HNTM, resulting in potent US-propulsion capabilities of HNTM-MoS_2_. The synergistic impact of the generated ROS and robust mechanical force demonstrates an antibacterial efficacy of 98.5% against methicillin-resistant *Staphylococcus aureus* (MRSA) after just 15 minutes of US treatment. In MRSA, this process results in intracellular DNA damage, heightened oxidative stress, and disturbances in purine metabolism, tryptophan metabolism, as well as pantothenate and CoA biosynthesis. Furthermore, possessing the capability to neutralize toxins, the RBC-HNTM-MoS_2_ composite effectively eradicates bone infections, mitigates bone loss and suppresses inflammation. This research introduces an innovative strategy for developing efficient sonosensitizers by leveraging piezoelectric-assisted sonocatalysis and enhancing US-propulsion capabilities.

Interface charge transfer is another method to reduce O_2_ into ^1^O_2_. The g-ZnN_4_-MoS_2_ composite was prepared *via* electrostatic interaction, where the Zn single-atom catalysts (g-ZnN_4_) exhibited excellent biocompatibility (Fig. 9b).^112^ Serving as a co-catalyst, MoS_2_ QDs offer abundant active sites that facilitate highly mobile charge transfer pathways, thus enhancing photoinduced charge carrier separation efficiency. Through the construction of heterogeneous interfaces, the g-ZnN_4_-MoS_2_ composite efficiently generates singlet oxygen (^1^O_2_) under ultrasound (US) irradiation. This is attributed to enhanced interface charge transfer and reduced O_2_ activation energy. The continuous release of Zn^2+^ at a safe concentration ensures the biological functionality of g-ZnN_4_-MoS_2_. Both i*n vitro* and *in vivo* research studies validate the exceptional sonocatalytic and osteoinductive capabilities of g-ZnN_4_-MoS_2_, effectively addressing osteomyelitis infected with MRSA.

### 4.3 Tuning the band structure for SDT

**Fig. 10:**
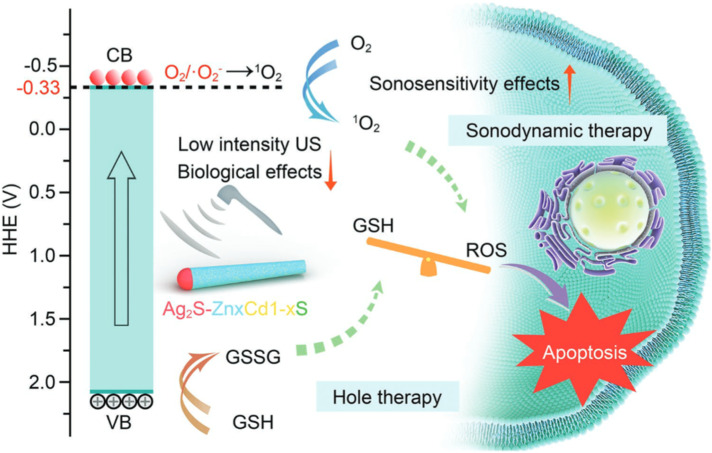
Illustration showing the synergistic anti-tumor treatment involving sonodynamic therapy (SDT) and the generation of oxidative holes utilizing designed Ag_2_S–Zn_*x*_Cd_1-*x*_S heteronanorods.^113^

Sonosensitizer-assisted SDT has shown great potential as a strategy for treating cancer. However, there is limited understanding regarding the specific regulations of sonosensitizer band structures in relation to oxygen within tissues. Researchers developed a range of hetero-semiconductor sonosensitizers using a doping technique that incorporates transition elements. This was done to finely adjust the band structure, particularly their reduction potentials, with the aim of investigating the relationship between reduction potentials and the production of ROS. Within the realm of diverse nanostructures, the introduction of transition elements through doping proves adept at flexibly adjusting band gaps and electronic energy levels across both conduction and valence bands. This presents a reliable approach for researching and selecting sonosensitizers with high efficiency that could be effectively stimulated under low-intensity ultrasound. Simultaneously, the US-activated oxidative holes exhibited the ability to convert glutathione (GSH) into glutathione disulfide (GSSG), thereby disrupting the redox equilibrium within tumor lesions and enhancing the response to oxidative stress conditions. The operational mechanism depicted in Fig. 10 is explained by the designed Ag_2_S–Zn_*x*_Cd_1-*x*_S heteronanorods.^113^ This study demonstrates the application of toxic reactive ROS produced by specifically designed hetero-semiconductor sonosensitizers for targeting tumors. The findings verify that the effectiveness of the treatment originates not only from tuning the band structure, but also from the distinct reduction potentials of the hetero-semiconductor sonosensitizers. Remarkable suppression of tumor growth was attained with a low ultrasound (US) intensity of 0.5 W cm^−2^, without causing temperature-related impacts or harm due to US irradiation. In addition, hetero-semiconductors present unique characteristics, including guidance *via* fluorescence imaging and responsiveness of reactive ROS to the enhanced acidity within the tumor microenvironment. These attributes further contribute to the improvement of therapeutic effectiveness.

## 5. Ferroptosis of two-dimensional transition metal sulfides

### 5.1 Ferroptosis of MoS_2_

**Fig. 11:**
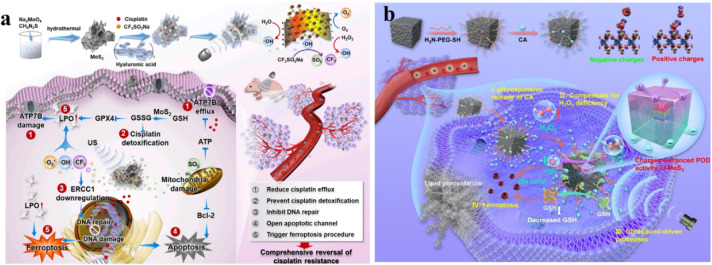
(a) Diagram illustrating the process of ˙OH, –CF_3_, and SO_2_ formation in HA@MoCF_3_Pt NPs and the potential mechanism for inhibiting cisplatin resistance under ultrasonic irradiation.^117^ (b) Schematic depiction of the enhanced catalytic activity for efficient antitumor therapy through positive and negative charge modulation in MoS_2_ POD.^119^

Over the past few years, there has been extensive research on piezoelectric materials, including BaTiO_3_, ZnO, BiFeO_3_, and MoS_2_, in various fields such as catalysis, pollutant degradation, energy transformation, and tumor eradication. This is attributed to their effectiveness in generating oxygen radicals, including radical ˙O_2_^−^ and radical ˙OH, coupled with excellent stability.^114–116^ Oxygen radicals, as opposed to ^1^O_2_, exhibit enhanced efficacy in hypoxic tumor environments, a crucial aspect for in-depth tumor treatment. Moreover, by the application of ultrasonication-triggered electron transfer, these piezoelectric materials not only generate oxygen radicals but also produce carbon radicals known for their exceptional stability. Drawing inspiration from piezo-catalysis, a proof-of-concept was established through the fabrication of an innovative nanoplatform activated by ultrasound, namely HA@MoCF_3_Pt (Fig. 11a).^117^ During ultrasound irradiation, MoS_2_ nanofibers (NFs) catalyze the breakdown of H_2_O_2_ and H_2_O, producing radical ˙OH and radical ˙O_2_^−^, respectively. Subsequently, radical ˙OH triggers the decomposition of CF_3_SO_2_Na into radical ˙CF_3_ and sulfur dioxide (SO_2_).^118^ Leveraging its nanoenzymatic properties, MoS_2_ NFs inhibit the detoxification of cisplatin by oxidizing GSH to GSSG. Simultaneously, the generation of radical ˙O_2_^−^, radical ˙OH, and radical ˙CF_3_ leads to the excessive generation of lipid peroxides (LPO) within the cellular membrane. The buildup of ROS and LPO induces ferroptosis and disrupts the efflux protein (ATP7B). Notably, radical ˙CF_3_ is demonstrated, for the first time, to enhance cisplatin damage by inhibiting the excision repair cross-complementation group 1 enzyme (ERCC1, a DNA repair enzyme). Additionally, SO_2_ generated subsequently initiates the apoptotic program in cisplatin-resistant tumors by downregulating the B-cell lymphoma-2 (Bcl-2) protein. Hence, the MoS_2_-based intelligent network, serving as a sonosensitizer, nanoenzyme, and initiator of ˙CF_3_ radicals, collaboratively overcomes cisplatin resistance. This is achieved by enhancing drug accumulation, diminishing DNA repair enzyme activity, and suppressing cisplatin detoxification, while promoting dual-mode cell death in tumor cells. Wang *et al.* introduced a novel discovery where the surface of piezoelectric materials generates both positive and negative charges under US conditions, leading to an augmented peroxidase-like (POD-like) activity of MoS_2_. Theoretical elucidation of this phenomenon can be achieved by considering the reduced binding energy between MoS_2_ and H_2_O_2_, coupled with the facilitated dissociation of H_2_O_2_. To engineer this sequential-functional nanocatalyst, they constructed a hybrid structure by depositing few-layer MoS_2_ nanosheets onto the surface of a prototypical microscopic piezoelectric material, T-BTO nanoparticles, (Fig. 11b).^119^ This hybrid structure is further integrated with a polyethylene glycol (PEG)-modified system responsive to changes in pH with cinnamaldehyde (CA) to obtain BTO/MoS_2_@CA. This tethered CA addresses the limitations of free CA molecules, including inadequate stability, short *in vivo* half-life, and systemic toxicity. Functioning as the primary catalyst, the released CA catalytically generates abundant H_2_O_2_ within the acidic tumor microenvironment. This elevation in H_2_O_2_ enhances tumor specificity while mitigating adverse effects. The ensuing catalytic interaction of H_2_O_2_ with the downstream BTO/MoS_2_ complex considerably enhances the POD-like activity, generating highly toxic ˙OH radicals under US exposure. Concomitantly, due to its affinity for sulfhydryl groups, the generated oxidative stress leads to glutathione (GSH) depletion, causing a disruption in redox homeostasis and ultimately inducing ferroptosis in tumor cells by diminishing peroxidase 4 (GPX4) expression. The combination of pH-responsive carbonic anhydrase (CA)-facilitated H_2_O_2_ self-generation, ultrasound-triggered heightened enzymatic activity, and glutathione (GSH) reduction disrupts the equilibrium of redox homeostasis. This combination of the BTO/MoS_2_@CA nanocatalyst effectively induces tumor ferroptosis with minimal adverse effects.

### 5.2 Ferroptosis of FeS_2_

**Fig. 12:**
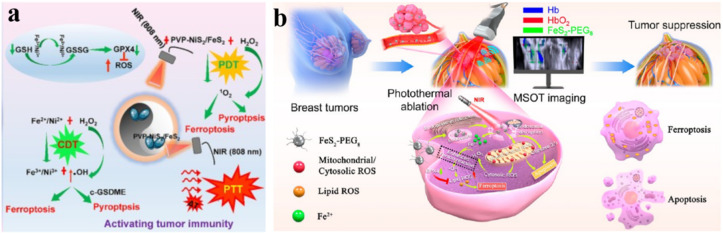
(a) Elucidating the mechanisms by which PVP-NiS_2_/FeS_2_ NPs achieve therapeutic effects through the integration of CDT/PDT/PTT therapy.^122^ (b) The FeS_2_-PEG_8_ system circulates systemically, passively targeting tumors *via* fenestrated endothelial vasculature through enhanced permeability and retention. After entering cells through electrostatic interactions and macropinocytosis, MSOT imaging is employed for visualizing and locating the primary tumor mass, followed by concurrent induction of apoptosis and ferroptosis through photothermal ablation.^125^

Considering the heterogeneity and complexity of the tumor microenvironment, effectively treating solid tumors using a single therapeutic approach remains challenging.^120^ The potential to address this challenge lies in the creation of multifunctional nanomaterial strategies for synergistic chemo-dynamic/photo-dynamic/photothermal therapy. Given the importance of ferroptosis in suppressing tumors, diverse nanomaterials have been crafted to selectively target the iron-dependent cell death pathway.^121^ Utilizing a simple hydrothermal process and subsequent calcination treatment, Xu *et al.* synthesized novel NiS_2_/FeS_2_ nanoparticles (NiS_2_/FeS_2_ NPs). These were further modified with polyvinyl pyrrolidone to improve their biocompatibility. The resulting PVP-NiS_2_/FeS_2_ NPs demonstrated the ability to elicit synergistic cancer therapy effects encompassing PTT, PDT, and CDT, all under single-wavelength NIR irradiation (Fig. 12a).^122^ Moreover, these nanoparticles possessed the capability to perform both magnetic resonance (MR) imaging and photoacoustic (PA) imaging. The photocatalytic nature of PVP-NiS_2_/FeS_2_ NPs was apparent through their ability to generate abundant singlet oxygen (^1^O_2_) under irradiation. Due to the presence of multivalent ions Fe^2+^/Fe^3+^ and Ni^2+^/Ni^3+^, PVP-NiS_2_/FeS_2_ NPs effectively induce the CDT effect, ferroptosis, and pyroptosis by generating a substantial amount of ˙OH. Furthermore, through the synergistic application of CDT, PTT, and PDT, activated by near-infrared light, PVP-NiS_2_/FeS_2_ NPs exhibit outstanding tumor elimination in subcutaneous 4T1 tumors in syngeneic BABL/c mice. Additionally, they inhibit tumor metastasis by suppressing the epithelial–mesenchymal transition (EMT) pathway. Therefore, PVP-NiS_2_/FeS_2_ NPs represent a promising candidate for therapeutic intervention based on CDT/PTT/PDT, further enhanced by their PA/MR imaging capabilities.

Triple-negative breast cancer (TNBC) is recognized for its heightened aggressiveness and poorer prognosis compared to different subtypes of breast cancer.^123^ Researchers developed the Ferroptosis Potential Index (FPI) by conducting thorough bioinformatic analyses, utilizing the expression profiles of genes that regulate ferroptosis.^124^ Notably, the findings indicated that TNBC exhibits a substantially higher FPI in comparison to non-TNBC counterparts, as observed in both human breast cancer cell lines and tumor tissues. As reported, the synthesized FeS_2_-PEG8 exhibits high power conversion efficiency (PCE up to 63.1%), serum stability, and photostability (Fig. 12b).^125^ This enables contrast-enhanced multispectral optoacoustic tomography (MSOT) imaging and noninvasive PTT in the biologically “transparent” NIR phototheranostic window of breast tissues. In an acidic microenvironment, FeS_2_-PEG8 releases redox-active iron(ii) species, increasing the labile iron pool (LIP). Under NIR irradiation, Fenton reactions are accelerated, leading to an increase level of radicals within the cells. Subsequently, ferroptosis is induced, concurrently inhibiting tumor growth and metastasis both *in vitro* and *in vivo*, surpassing caspase 3/9-dependent apoptosis. Therefore, this approach holds promise in overcoming apoptosis resistance. Local near-infrared photothermal intervention is minimally invasive, providing clinicians with an opportunity to destroy unresectable primary and metastatic TNBC tumors under image guidance. This approach holds significant promise in both standalone and combination therapies.

## 6. Conclusions and outlook

In summary, the diverse applications of transition metal sulfides across various fields hold undeniable promise and abundant potential. The exploration of 2D-TMSs has paved the way towards pioneering advancements in technology, medicine, and cancer therapy. 2D-TMSs, as versatile materials, offer a broad range of applications and a promising future. From advancing technological innovation to revolutionizing healthcare and cancer therapy, their trajectory underscores their immense significance in the realm of scientific and technological development. As we continue to delve deeper into the unique properties and capabilities of 2D-TMSs, we can anticipate the emergence of even more transformative innovations in the years ahead. Here are some perspectives on the prospects of 2D-TMSs in these therapeutic methods:

(1) PDT:

Targeted and specific treatment: future developments may focus more on improving the targeted and specific aspects of photodynamic therapy to reduce the impact on surrounding normal tissues. This could involve 2D-TMSs design and more precise treatment planning.

Tunable photosensitizer design: surface modification and functionalization of 2D-TMSs allow the control of their photosensitizing properties. This may involve adjusting the wavelength absorption range, photostability, and targeting capability.

(2) PTT:

Application of nano-carriers: integrating 2D-TMSs into nano-carriers can enhance their *in vivo* distribution and targeting, improving the local effects of photothermal therapy.

Development of thermosensitive nanomedicines: further research and development of 2D-TMS nanocarriers capable of releasing thermosensitive drugs *in vivo* could achieve localized and targeted drug release, enhancing therapeutic effects.

Integration of photothermal therapy and immunotherapy: combining photothermal therapy with immunotherapy could enhance the immune response, achieving a more comprehensive therapeutic effect.

(3) SDT:

Acoustic response characteristics: modulating the acoustic response properties of 2D-TMSs can make them more significant in sonodynamic therapy. This may involve the material's acoustic sensitivity and its efficiency in converting acoustic wave energy.

Multifunctional sonodynamic therapy: combining the acoustic properties of 2D-TMSs in combination with alternative treatment modalities, such as photodynamic therapy or photothermal therapy, can achieve multifunctional sonodynamic therapy to enhance therapeutic effects.

(4) Ferroptosis:

Iron ion regulation: transition metal elements in 2D-TMSs can influence cell ferroptosis by regulating the presence and release of iron ions. Future research may further explore this mechanism.

In-depth understanding of the ferroptosis mechanism: in-depth research into the mechanism of ferroptosis, including the regulation of intracellular iron ions and lipid peroxidation, will contribute to a better understanding of this form of cell death.

Discovery of new targets and drugs: further exploration and discovery of new targets and drugs that can regulate ferroptosis may improve treatment outcomes for certain cancers or diseases. Research on ferroptosis is evolving towards more targeted and personalized approaches to minimize damage to normal tissues.

Design of nano-carriers: combining 2D-TMSs with nano-carriers having iron-regulating functions can achieve more precise ferroptosis therapy, reducing the impact on surrounding normal tissues.

Overall, 2D-TMSs hold broad application prospects in the mentioned therapeutic methods. Future research will focus on optimizing material properties, improving targeting capabilities, implementing multimodal therapies, and exploring new treatment strategies to enhance efficacy and minimize side effects in patients.

## Data availability

No data were used for the research described in the article.

## Author contributions

Fei Luo: methodology, writing – original draft. Gang Zhou: formal analysis, writing – review & editing. Youfu Wang: formal analysis, writing – review & editing. Shaohua Song: data curation, formal analysis, funding acquisition. Hao Liu: conceptualization, data curation, writing – original draft, writing – review & editing.

## Conflicts of interest

The authors declare that they have no known competing financial interests or personal relationships that could have appeared to influence the work reported in this paper.
